# Retinal Ganglion Cell Death as a Late Remodeling Effect of Photoreceptor Degeneration

**DOI:** 10.3390/ijms20184649

**Published:** 2019-09-19

**Authors:** Diego García-Ayuso, Johnny Di Pierdomenico, Manuel Vidal-Sanz, María P. Villegas-Pérez

**Affiliations:** Departamento de Oftalmología, Facultad de Medicina, Universidad de Murcia, and Instituto Murciano de Investigación Biosanitaria Virgen de la Arrixaca (IMIB-Virgen de la Arrixaca), 30120 Murcia, Spain; johnnydp@um.es (J.D.P.); manuel.vidal@um.es (M.V.-S.)

**Keywords:** cones, retinal degeneration, retinal remodeling, retinal ganglion cells, axonal compression, neurovascular alterations

## Abstract

Inherited or acquired photoreceptor degenerations, one of the leading causes of irreversible blindness in the world, are a group of retinal disorders that initially affect rods and cones, situated in the outer retina. For many years it was assumed that these diseases did not spread to the inner retina. However, it is now known that photoreceptor loss leads to an unavoidable chain of events that cause neurovascular changes in the retina including migration of retinal pigment epithelium cells, formation of “subretinal vascular complexes”, vessel displacement, retinal ganglion cell (RGC) axonal strangulation by retinal vessels, axonal transport alteration and, ultimately, RGC death. These events are common to all photoreceptor degenerations regardless of the initial trigger and thus threaten the outcome of photoreceptor substitution as a therapeutic approach, because with a degenerating inner retina, the photoreceptor signal will not reach the brain. In conclusion, therapies should be applied early in the course of photoreceptor degeneration, before the remodeling process reaches the inner retina.

## 1. Introduction

Retinal remodeling is a term used to describe the events initiated by photoreceptor stress and death in retinal degenerations of different etiologies [1,2]. These events occur post-photoreceptor degeneration and include changes in gene expression, neuronal neuritogenesis and death, migration of retinal cells, vessel displacement and rewiring of some circuitries [1,3,4,5,6,7,8,9,10,11,12,13,14,15]. It is believed that remodeling is a negative plasticity of the retina because it impedes possible rescue strategies [2].

Many inherited, acquired or induced retinal diseases cause photoreceptor degeneration and therefore, photoreceptor degenerations are at present one of the leading causes of irreversible blindness in the world [16,17]. The most frequent acquired cause of photoreceptor degeneration is age-related macular degeneration (AMD), characterized by photoreceptor and retinal pigment epithelium (RPE) cell degeneration in the central retina [17]. This disease is becoming a major health concern due to the increase in life expectancy [17]. Inherited photoreceptor degenerations are not as frequent as AMD but are important because they cause irreversible blindness at working ages [18] and thus represent a worldwide health problem as well [19]. The most common form of inherited retinal degeneration is retinitis pigmentosa (RP) with a prevalence of around 1 in 4000 individuals [20]. This disease is the consequence of gene defects that generally affect the photoreceptors and the RPE [20,21,22,23,24]. Most forms of RP present primarily with rod loss, which causes night blindness at the onset of the disease, but later in the disease there is secondary cone loss, and therefore blindness [20,24]. The reasons why rod degeneration causes cone degeneration are poorly understood and have been interpreted by various authors [24,25,26,27]. However, there are other forms of RP that present primarily with cone and rod loss, as there are forms affecting both rods and cones or the RPE [27].

For many years, it was thought that the diseases that caused photoreceptor loss affected only the external retina, leaving the internal retina relatively intact. It was not until very recently that research has shown that photoreceptor loss initiates a unavoidable chain of events, known as retinal remodeling, that cause alterations of the inner retina [2,3,4,6,9,10,13,28], which, in its final state, and after a complete loss of photoreceptors, causes retinal ganglion cell (RGC) loss. Although the main objective of retinal degeneration research has been to develop therapies that slow or prevent photoreceptor death or that replace photoreceptors to restore vision [21,29,30,31], it also seems important, from a therapeutic point of view, to know what the effect of retinal remodeling is on the RGCs, the neurons that transmit the information to the brain [4,6,9,10,13]. In fact, severe retinal remodeling could threaten the outcome of the treatments aimed to replace the dead photoreceptors, since the presence of functional RGCs is essential for the transmission of the visual information to higher brain centers.

The general features of retinal changes following photoreceptor loss have been widely studied and seem to be common to all photoreceptor degenerations, independently of the strain or the etiology of the degeneration [2,4,6,9,10,11,13,15,28] and have also been described in human retinal degenerations [32,33]. The process of retinal degeneration comprises four different phases: (i) primary photoreceptor stress and loss; (ii) secondary photoreceptor degeneration and involvement of microglia, Müller and RPE cells; (iii) tissue remodeling, including neuronal rewiring, neuronal death and disorganization of the retina [1]; and (iv) progressive neurodegeneration [34]. Remodeling is considered negative, because it culminates with the death of further retinal neurons [9,10,13,34]. In the next section, we will briefly review the most important events of retinal remodeling for the later affectation of RGCs.

## 2. A Quick Look at the Early Stages of Retinal Remodeling

All photoreceptor degenerations seem to evolve similarly independently of the initial event [1,13,15,34]. They cause photoreceptor death by apoptosis [10,35], morphological and topographical changes in the surviving photoreceptors [27,36,37,38,39,40,41], deafferentation of bipolar cell populations [28], and retinal glial cell activation [27,35,41,42].

**Figure 1:**
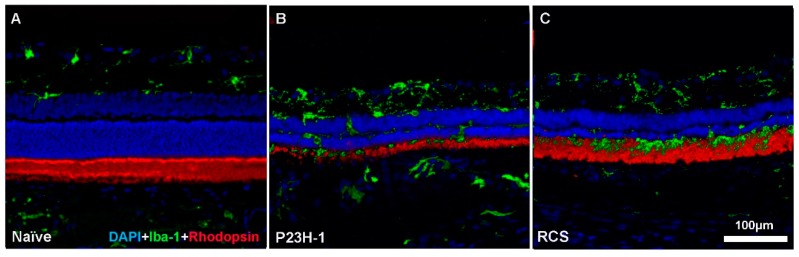
Rod degeneration and microglial cell responses. Microphotographs of representative retinal cross sections from a naïve SD rat (**A**) showing a normal retina, a retina from a P23H-1 rat (**B**) and a retina from an RCS rat (**C**) in the early stages of retinal degeneration. Rod outer segments appear in red and microglial cells in green. In the P23H-1 rat retina, rhodopsin expression was clearly affected; however, in the RCS rat, rhodopsin expression is not altered since the retinal pigment epithelium (RPE) cells are not phagocytising the rod outer segment debris. In both experimental retinas, microglial cells were activated and migrated from the inner to the outer retinal layers.

**Figure 2:**
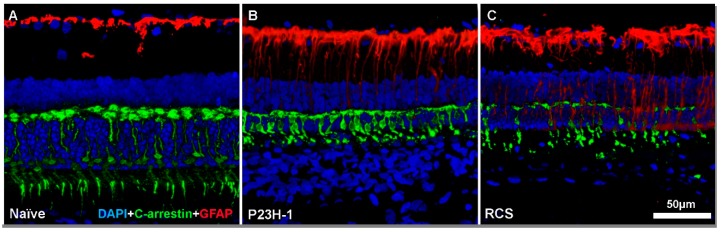
Cone degeneration and macroglial cell response. Microphotographs of representative retinal cross sections from a naïve SD rat (**A**) showing a normal retina, a retina from a P23H-1 rat (**B**) and a retina from an RCS rat (**C**) in the early stages of retinal degeneration. Cone outer segments appear in green and macroglial cells (GFAP) in red. In both P23H-1 rats and RCS rats, cones degenerate and lose their typically elongated morphology. Moreover, GFAP overexpression in astrocyte and Müller cells could be seen.

Among all the events that occur in the early phases of retinal remodeling, glial activation may play an essential role in the subsequent affectation of RGCs. During the course of photoreceptor degeneration, glial cells are mobilized to the outer retinal layers [22,27,35,41,42,43]; microglial cells become activated and migrate to phagocytose dying photoreceptors [27,35] (Figure 1) and Müller cells become hypertrophic, fill the space left by dead photoreceptors and form a gliotic seal [32,35,41,42,43,44,45,46,47] (Figure 2). Specifically, it has been documented that glial activation is a common theme in photoreceptor degenerations regardless of their aetiology [35,41,48,49], and that treatments that inhibit microglia [27,43,49] or macroglia [50] can influence the course of the disease. This is probably one of the principal hallmarks of the evolution of photoreceptor degenerations [28,51] and will be important for RGC affectation, because if the gliotic seal is not complete, there might be gaps through which RPE cells invade the retina [4,9,10,28]. Moreover, the gliotic seal or glial scar may have detrimental effects by impeding regenerative processes and thus contributing to neurodegeneration and further retinal remodeling [52].

The end-stage of retinal remodeling induces changes in the inner retina that include neuronal cell death, cell migration, and rewiring with abundant new synaptic connections resulting in corrupt visual circuitry [1,28,34]. This rewiring could be an attempt from the remaining neurons to survive. This is supported by the hypothesis that excitatory inputs are imperative for neuronal survival [28]. At this point, retinal rescue is not possible.

There are two main types of neurons in the inner retina: amacrine cells and RGCs. Amacrine cells remain relatively stable but can undergo molecular changes and connect to a new network of aberrant synaptic connections. However, RGCs are undoubtedly the most relevant neurons in the inner retina since their axons form the optic nerve and send the visual information to the visual cortex in the brain in a process that is essential for being able to see.

## 3. Retinal Remodeling and Retinal Ganglion Cells

Even though it has been documented that RGCs remain stable during retinal remodeling [15,53,54,55], most of these studies have evaluated RGC morphology [53,54,55,56]. However, little is known to date about the possible consequences of retinal remodeling in RGC axonal transport or in RGC survival through a detailed study of the RGC population. For that, a reliable method is needed to identify (i.e. retrograde labeling, phenotypic markers) and quantify (i.e. retinal sections, whole mounts) RGCs. It is important that the method of identification leaves no doubts that it is specifically labelling RGCs and not amacrine cells [57,58,59,60] and that the counting technique can quantify every single RGC present in the retina [57,58,59,60,61,62,63].

Several techniques have been used to identify RGCs, but not all have been shown to specifically and accurately identify RGCs. For example, βIII tubulin, γ-synuclein and islet-1 identify both RGC and amacrine cells [60,64], and whilst Thy-1 has been proposed to specifically label RGCs, it is downregulated after injury and therefore it is not a good marker for studies assessing RGC death and neuroprotection [59,60]. RNA-binding protein with multiple splicing (RBPMS) is suggested to specifically label RGCs, however, there are few studies using it and it is important to study its suitability, specially under RGC degeneration [60].

Our group has broad experience in the study of the RGC population (reviewed in [65]) and we have used different methodological techniques to study the consequences of remodeling on RGCs in inherited and induced rat models of photoreceptor degeneration. We favor the use of the retrograde tracer fluorogold (FG; [57]) and the detection of the transcription factor Brn3a [58]. FG is an actively retrogradely transported tracer that, when applied onto both superior colliculi, labels approximately 98% of RGCs in the rat retina [57,63]. Brn3a is a transcription factor specifically expressed in RGCs and therefore can be immunodetected as a nuclear marker [58]. It labels approximately 96% of RGCs [58] and has been proposed as an indirect indicator of the functional status of the RGCs [66,67,68]. We have developed specific cell counting subroutines that we have shown to be reliable and reproducible methods to quantify whole populations of FG labeled RGCs and Brn3a immunodetected RGCs [57,58,62,63].

We have used these self-developed techniques to analyse the late effects of photoreceptor degenerations in the retina [69,70] using two well-known models of inherited retinal degeneration: the P23H-1 rat and the Royal College of Surgeons (RCS) rat, and a model of light-induced retinal degeneration in albino and pigmented rats. The inherited models faithfully represent the human disease, because P23H rats suffer from one of the most frequent mutations observed in human retinal degeneration [71] and RCS rats suffer from a mutation in the *MERKT* gene (also observed in some RP patients) which impairs the ability of RPE cells to phagocytose [71]. Light-induced retinal degeneration models were first described by Noell [72] and have been used for years to replicate human retinal degeneration. Different light-induced retinal degeneration models have been devised, and we have developed our own method using cold white light, which has been previously described in detail [6,10,38,39]. Light-induced retinal degeneration models have been shown to mimic degenerative retinal diseases [70], especially the late stages of age-related macular degeneration [8]. There are still significant gaps in the available animal models of retinal degeneration; in particular, better models of AMD are needed. However, these animal models are useful for studying the pathophysiology of retinal degeneration [71], as well as the efficacy and safety of different present and future treatments [23,71,73].

Our studies show that long after the occurrence of photoreceptor degeneration, there is a decrease in the mean number of RGCs in both inherited and induced retinal degenerations [3,4,6,9,10,12,13,37,38]. So, the question arises: what is the cause of the RGC death during retinal remodeling? We also have extensive experience in the study of the retinal nerve fiber layer (RNFL) by immunodetecting the phosphorylated high-molecular-weight subunit of the neurofilament triplet (pNFH), which in homeostasis is expressed in the intra-retinal RGC axons [71,72,74,75]. Changes in the expression pattern of pNFH is an early hallmark of RGC degeneration, and thus this immunodetection is a very good tool to analyze the course of RGC pathological responses to photoreceptor diseases [9,10,74,76,77].

The classic findings in the funduscopic imaging of human RP patients seen in advanced stages of the disease are bone spicules [2] and optic nerve pallor [78]. Optic nerve pallor is believed to be the result of RGC death [78,79], and bone spicules are formed by migration of RPE cells associated with retinal vessels [2,78]. This RPE invasion of the retina may be permitted by gaps in the glial seal [28], and we have proposed that these cells migrate in response to the proximity of the retinal vessels that come in contact with the RPE [4,5,28,80]. The migration of the RPE cells on the retinal vessels causes vessel displacement, formation of “subretinal vascular complexes” and axonal strangulation by the inner displaced retinal vessels which is ultimately responsible for RGC death [4,5,9,10]. It is important to note that vascular changes will not occur until severe photoreceptor death has occurred [4,5,9,10,11,13,81] and is prevented if a significant number of photoreceptors remain alive [82].

**Figure 3:**
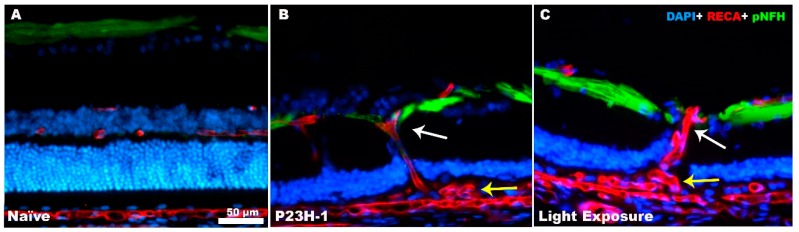
Retinal remodeling causes axonal and vascular changes. Microphotographs of retinal cross sections from a naïve SD rat showing a normal retinal structure (**A**), the retinal structure of a P23H-1 rat (**B**) and the retinal structure of a light-exposed SD rat (**C**) after the complete loss of photoreceptors. Retinal ganglion cell (RGC) axons appear in green labelled with anti-neurofilament antibodies (pNFH), the blood vessels in red labelled with anti-rat endothelial cell antigen (RECA) and the nuclei in blue labelled with DAPI. In B and C, blood vessels are running vertically in the retina (white arrow) because they are dragged to the subretinal vascular complexes that appear between the RPE and Bruch’s membrane (yellow arrow) with the inner retinal vascular plexus. RGC axons are displaced by the vessels, which will eventually cause RGC death.

Thus, to understand the causes of RGC death during retinal remodeling, it is important to know the progressive sequence of changes in the vascular supply to the retina that occurs secondary to photoreceptor loss [81]. As a consequence of photoreceptor loss, there are various changes that will precipitate the vascular changes: (i) an increase in retinal hyperoxia, which presumably suppresses the expression of different growth factors such as vascular endothelial growth factor (VEGF) [24,82,83]; (ii) a breakdown of the blood–retinal barrier [6,10]; (iii) the outer vascular plexus approaches the RPE cell layer [5]. These events presumably stimulate the migration of the RPE cells that envelop the retinal vessels to form “subretinal vascular complexes” composed of tortuous displaced vessels [6,10] (Figure 3). Although some authors have proposed that these complexes are the result of neovascularization from the choroid, we have proposed that these vessels belong to the outer retinal plexus and that their change in phenotype is due to the influence of the RPE [5,6], and this idea is supported by the fact that Bruch´s membrane is intact in these retinas [4].

**Figure 4:**
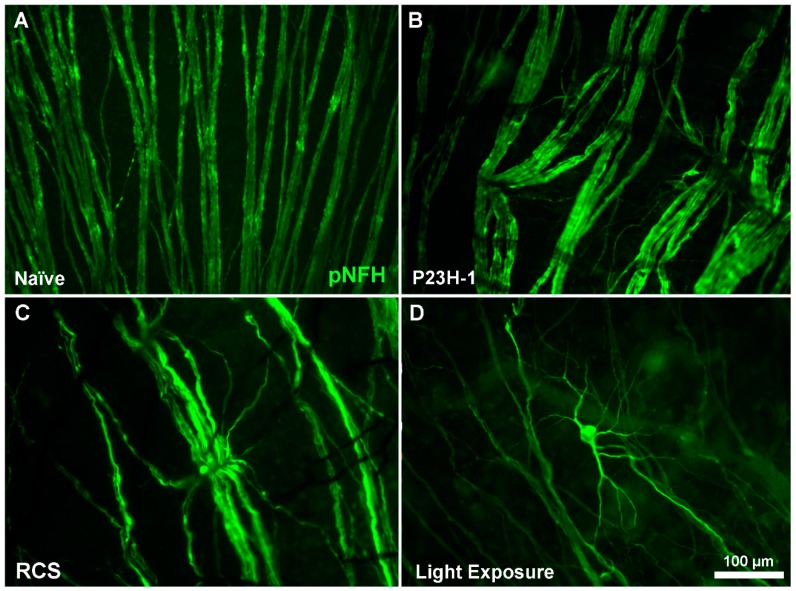
RGC axonal alterations. Magnifications from flat mounted retinas from a naïve SD rat (**A**), a P23H-1 rat (**B**), an RCS rat (**C**) and a light-exposed SD rat (**D**) after a complete loss of photoreceptors. RGC axons appear in green labelled with anti-neurofilament antibodies (pNFH). In the degenerating retina, the linear trajectory of RGC axons is disrupted and, in addition, some RGC bodies and their proximal dendrites appear labelled with pNFH, indicating RCG degeneration.

The above-mentioned vascular complexes are in communication with the vessels of the inner vascular plexus of the retina [4,5,6,9,10,81,84] (Figure 3). The migration of RPE cells along the retinal vessels drag the inner retinal vessels which, in turn, compress the RGC axons [4,5,6,9,10,11] and cause RGC axonal interruption and death [5,6,9,10] (Figure 3 and Figure 4). Neurovascular alterations have also been described in some of the most devastating diseases of the CNS affecting humans, such as Alzheimer´s disease and other age-related diseases [85,86,87,88]. Interestingly, it has been recently proposed that in the end-stage of retinal remodeling, progressive neurodegeneration resembles the proteinopathies observed in the abovementioned CNS diseases [34]. The location of the retina in the posterior pole of the eye makes it an easily accessible portion of the CNS with some unique advantages for conducting studies targeting neurodegeneration and neuroprotection. This excitable tissue is consequently excellent for studying and understanding these neurovascular alterations and, therefore, the progression of various CNS diseases.

Neurovascular alterations in the degenerated retina following photoreceptor loss are first observed because the normal linear trajectory of the RGC axons (Figure 4), which normally diverge from the optic nerve, is disturbed at the axon–vasculature crossing points. At these points, RGC axons show distorted non-linear trajectories (Figure 4) caused by the dragging and compression of the axons by retinal vessels [3,4,5,6,9,10,11,13,89] (Figure 1 and Figure 4). These strangulations are first seen in the ventral retina in the P23H-1 and RCS rats [9] and in the dorsal retina after light exposure [10]. These areas coincide with the regions in which photoreceptors are first lost [9,10,39]. As degeneration progresses, this phenomenon becomes more severe and these degenerating areas are seen all over the retina and in all of the studied models [9,10,11,39]. At the oldest ages analyzed, signals of axonal transport interruption and axonal transection such as axonal bulbs and wandering axons are seen [9,10,11,39] (Figure 4). Interestingly, in long-term post-photoreceptor degeneration, some RGC bodies and dendrites became pNFH^+^ [9,10,11,39] (Figure 4).

Similar alterations in the pNFH expression pattern have been observed following other retinal degenerations such as optic nerve transection [71,72], crush [74,76,90], elevation of intraocular pressure [91,92] or taurine depletion [75]. These interesting events, and specifically pNFH expression in the RGC bodies and their dendrites, have been proposed as a feature of RGC degeneration [62], particularly with pathological processes associated with axonal damage and RGC stress [74,75,76].

**Figure 5:**
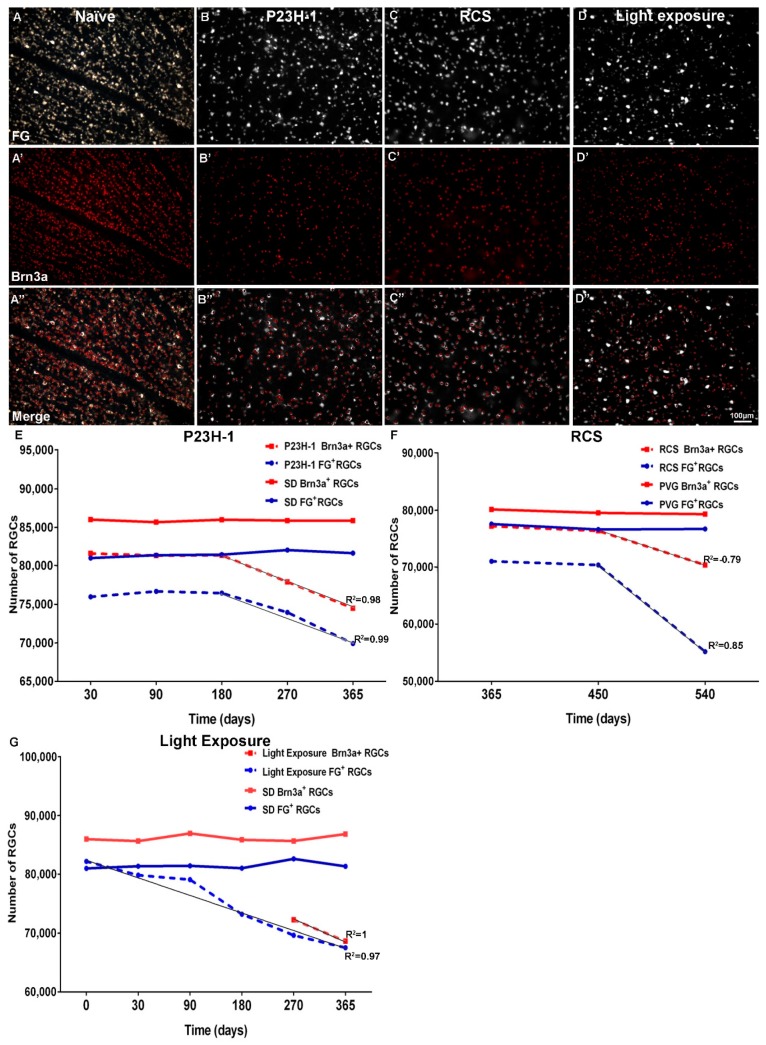
RGC loss following photoreceptor degeneration. Microphotographs from a representative naïve SD rat retina and a P23H-1, RCS and light-exposed retina showing fluorogold (FG)+RGCs (**A**, **B**, **C**, **D**), Brn3a+RGCs (**A’**, **B’**, **C’**, **D’**), merged images (**A’’**, **B’’**, **C’’**, **D’’**), and X, Y graphs showing the evolution of the RGC populations (Y axis) with age or time (X axis) in naïve SD and P23H-1 rats (**E)**, RCS and Pievald Viro Glaxo (PVG) rats (**F**) and in naïve SD and light-exposed SD rats (**G**). Data are from García-Ayuso et al. [9,10,11].

Surprisingly, when studying the population of RGCs, we found that the P23H-1 rat had fewer RGCs than their homologous Sprague–Dawley (SD) counterparts [9] (Figure 5). Since P23H-1 rats are derived from the SD strain, we expected to find similar numbers between both strains. So, the question arose: would the rhodopsin mutation suffered by these rats affect the development of the inner retina? According to a previous study that evaluated retinal blood vessel formation during development in two different animal models of retinal degeneration, photoreceptor loss during vascular development affects the formation of the deep vascular plexus, which supplies the inner retina [82]. In our opinion, it may be the cause of the lower number of RGCs found in this strain [9].

It has been shown in detail that the consequence of the above-described axonal damage is RGC death [6,9,10,11], and that in the RCS rat, this is accompanied by axonal transport impairment [11,93,94]. It has also been shown that RGC death is a late event in retinal degeneration, because it does not occur in the early stages of photoreceptor loss (not until nine months of age in the P23H-1 rat [9], six months of age in the RCS rat [3,4,11] or until 90 days after light exposure in the albino rat [10]). However, it has been shown that following photoreceptor degeneration, there is a progressive loss of RGCs. It has been documented that at post-natal day (P) 365, P23H-1 rats have lost 14% of the RGCs [9] (Figure 5). One year after light exposure, albino rats have lost 18% of their RGCs [10] (Figure 5), and 17–19 months after light exposure, pigmented rats have lost 31% of RGCs. At P540, RCS rats have lost 29% of their RGCs [11] (Figure 5). 

In order to assess whether RGC loss in these models was due to axonal transport impairment and/or due to RGC death, tracing and immunodetection were combined. RGCs were traced one week before sacrifice with FG, a molecule that is retrogradely transported from the superior colliculi to the RGC somas. In these traced retinas, Brn3a, a marker of RGC viability, was immunodetected (Brn3a^+^RGCs). Thus, a decrease in traced RGCs (FG^+^RGCs) but not of Brn3a^+^RGCs, would indicate an axonal transport impairment. The loss of Brn3a^+^RGCs (RGC death) was similar to the loss of FG^+^RGCs (axonal impairment or RGC death) in the P23H-1 rat [9] and long term (270 days or more) after light exposure [10], amounting to a loss of 13% and 19% of their total population, respectively (Figure 5). However, in 540 day old RCS rats, the total number of Brn3a^+^RGC (loss of 12%) was higher than the number of FG^+^RGCs (loss of 29%), suggesting an axonal transport alteration also in this strain [11] (Figure 5; data not available for light exposure or RCS rats). Unfortunately, combining both methods, we only have data up to 365 days of life for P23H-1 rats or after light exposure, respectively [9,10] and from 365 days of life for RCS rats [11]. In spite of this fact, we observed that in the three models, there was a linear decrease in RGCs with a negative slope at older ages. The slope of regression lines for Brn3a^+^ and FG^+^ RGCs loss was similar in the P23H-1 rats (Table 1; Figure 5) and after light exposure (Table 1; Figure 5), with a calculated daily loss of 35 and 41 FG^+^RGCs, respectively, and of 37 and 38 Brn3a^+^RGCs, respectively, up to day 365 (the last time analyzed). However, in very old RCS rats (450 to 540 days) the loss of FG^+^RGCs was greater than the loss of Brn3a^+^RGCs RGCs (Table 1; Figure 5), with a daily loss of 91 and 32 RGCs, respectively. Thus, it appears that in the RCS rat, RGC loss seems to be preceded and/or accompanied by an impairment of axonal transport. However, these results do not allow us to ascertain whether retinal degeneration in the other studied models (P23H-1 and light exposure) may also be accompanied by an axonal transport impairment at older ages such as those analyzed for RCS rats. 

We and other authors have also found similar axonal–vascular alterations and RGC death in another animal model of inherited photoreceptor degeneration, the rd-1 mice, long term after photoreceptor degeneration [31,89]. In this strain, the cause of RGC death seems to also be axonal compression by the retinal vessels [89]. However, we cannot rule out if there could be other causes for RGC death in animal models of photoreceptor degeneration such as those observed in photoreceptor death, i.e. oxidative stress [95,96,97] or others such as cytopathologies, debris exocytosis, protein aggregation or even autophagy [33,98,99].

However, other studies performed in RCS rats [100] and in mice have failed to show RGC loss following photoreceptor degeneration [53,56,101]. These conflicting results may be explained because the RGC population was analyzed too early (before a complete loss of photoreceptors had occurred [100,101]), which is a prerequisite for axonal compression and the sensibility of the techniques applied to identify and/or quantify the RGC population that occur in localized sectors, which could be missed by other authors [53]. Studies performed in humans have documented RGC loss in human patients with photoreceptor degenerations [102,103,104,105,106,107,108]. 

This fact is critical for the successful implantation of photoreceptor transplants or protheses [80,109,110,111], especially in the very advanced stages of these diseases where extensive retinal remodeling leads to RGC death. In these patients, transplants or prostheses will not succeed because in a retina without RGCs, the visual signal will not reach the brain [3,4,6,9,10,11]. It could be argued that although there is evidence for RGC loss, a significant number of them remain in the retina. However, RGC loss is not the only drawback for these treatments to work properly; it is also necessary that the neuronal circuit remains normal so that synaptic connections can be created [80]. In line with this, it has been documented that retinal remodeling is accompanied by neuronal circuit corruption [31,112,113], questioning the possibility of the inner retinal neurons receiving synaptic inputs. Interestingly, a recent study has proposed that retina exhibit plasticity to re-establish some of the lost synaptic contacts following rod replacement [114]. However, the treatment was applied before complete photoreceptor loss and therefore further studies are needed to clarify this fact. In addition, the glial seal formed by Müller cells may also contribute to the failure of attempts to replace lost photoreceptors [52,106], for example limiting the migration of transplanted cells [113] and perhaps the creation of new synaptic connections.

In retinal degenerations, the integrity and function of photoreceptors is preserved for some time, suggesting that if we could slow down or stop their degeneration, we might be able to prevent secondary RGC loss. Consequently, research efforts have focused on the development of new pharmacological therapies to decrease photoreceptor loss, and not just on therapies to replace lost photoreceptors [23,115,116]. Many treatments such as trophic factors, anti-apoptotic drugs, antioxidant drugs, anti-inflammatory drugs, or gene therapies, among others, have been proposed to have beneficial effects on retinal degenerations [116,117,118,119,120,121]. For example, intravitreal (IVI) injections of anti-VEGF antibodies have been documented to slow the progression of some forms of AMD [122]. Concretely, in rodent models, recent publications have documented that IVI of the trophic factors basic fibroblast growth factor (FGF2) and ciliary neurotrophic factor (CNTF), and systemic administration of minocycline decreases photoreceptor loss [27,43]. These treatments could help to increase the time window in which therapies proposed to replace photoreceptors can be administered. However, intravitreal delivery has some potential risks that could limit its beneficial effects [123] and other administration routes should be explored.

## 4. Concluding Remarks and Future Directions

A common end for all retinal degenerations is that, with time, there is remodeling of the inner retina that results in RGC axonal strangulation, axonal transport alteration and RGC death. However, this fact has been missed in retinal degeneration literature for years, probably due to the lack of studies analyzing aged animals, thus allowing retinal remodeling to reach the innermost layers of the retina, and using techniques of RGC identification to label and quantify the RGC population. This inner retinal remodeling and its consequence (RGC death) could threaten the potential approaches to treat retinal degeneration using photoreceptor substitution, as only RGCs are capable of sending visual information to the brain. Thus, it is sensible to propose that retinal degeneration therapies should be applied in the early stages of photoreceptor degenerative diseases before the remodeling process reaches the inner retina.

## Figures and Tables

**Table 1:** Slope and correlation coefficients (R^2^) of each straight line from Figure 5.

Experimental Model	Identification Technique	Slope	R^2^
P23H-1	FG	−35.45 ± 4.23	0.98
	Brn3a	−37.12 ± 0.69	0.99
RCS	FG	−91.29 ± 46.44	0.79
	Brn3a	−32.09 ± 16.39	0.85
Light Exposure	FG	−41.45 ± 3.3	0.97
	Brn3a	−37.99	1
